# Engineering CAR T Cells to Target the HIV Reservoir

**DOI:** 10.3389/fcimb.2020.00410

**Published:** 2020-08-13

**Authors:** Wenli Mu, Mayra A. Carrillo, Scott G. Kitchen

**Affiliations:** Division of Hematology and Oncology, Department of Medicine, University of California, Los Angeles, Los Angeles, CA, United States

**Keywords:** HIV, latency, chimeric antigen receptor, gene therapy, LRA (latency reversing agents), immunotherapy, viral reservoir

## Abstract

The HIV reservoir remains to be a difficult barrier to overcome in order to achieve a therapeutic cure for HIV. Several strategies have been developed to purge the reservoir, including the “kick and kill” approach, which is based on the notion that reactivating the latent reservoir will allow subsequent elimination by the host anti-HIV immune cells. However, clinical trials testing certain classes of latency reactivating agents (LRAs) have so far revealed the minimal impact on reducing the viral reservoir. A robust immune response to reactivated HIV expressing cells is critical for this strategy to work. A current focus to enhance anti-HIV immunity is through the use of chimeric antigen receptors (CARs). Currently, HIV-specific CARs are being applied to peripheral T cells, NK cells, and stem cells to boost recognition and killing of HIV infected cells. In this review, we summarize current developments in engineering HIV directed CAR-expressing cells to facilitate HIV elimination. We also summarize current LRAs that enhance the “kick” strategy and how new generation and combinations of LRAs with HIV specific CAR T cell therapies could provide an optimal strategy to target the viral reservoir and achieve HIV clearance from the body.

## Introduction

HIV-specific CD8+ cytotoxic T lymphocyte (CTL) response plays a critical role in limiting the virus replication *in vivo* by recognizing viral antigens presented by human leukocyte antigen (HLA) class I and killing infected cells. However, the CTL response fails to durably control HIV replication in the absence of combination antiretroviral therapy (cART) (Jones and Walker, 2016). Intriguingly, very rare HIV-infected individuals, called “elite controllers,” are able to spontaneously control and suppress viral replication in the absence of cART. Elite controllers exhibit the core feature that defines a HIV “functional cure”: a long-term drug-free viral remission. There is compelling evidence from large genetic and functional immunology studies that robust CTL responses and protective HLA alleles are crucial for the natural control of HIV-infection (International et al., 2010; Walker and Yu, 2013). Even though natural CTL responses are imperfect and ultimately fail to clear the virus, they still drive partial control of viremia and, in the rare cases of elite controllers, is the dominant component of immune defense in successful long term suppression of viral replication. It is clear that a strong cellular immune response is essential in suppressing the virus and would be an essential component in therapeutic attempts to clear the virus from the body.

Despite the current cART to delay disease progression and prolong life expectancy, HIV remains to be an incurable disease for most. The inability for the host immune system to clear HIV from the body is partially due to the reduced present or absent viral antigen expression on latently infected CD4+ T cells that harbor integrated replication-competent virus (viral reservoir) that contribute to viral rebound once ART is discontinued (Churchill et al., 2016). Thus, one strategy that proposes to target the viral reservoir is referred to as “kick and kill” (also known as “shock and kill”) which postulates that inducing the virus from these latently infected cells (kick or shock) will facilitate “killing” by HIV mediated cell death or by the surrounding immune surveillance and lead to a clearance of the viral reservoir (Kim et al., 2018). However, clinical trials applying this strategy using latency reversal agents (LRAs) came short of promising results (Rasmussen et al., 2014; Spivak et al., 2014; Sogaard et al., 2015), suggesting that natural CTLs appear incapable of clearing this reservoir even after reactivating antigen expression. Although new strategies are improving the “kick” to induce virus, other studies have highlighted reasons for lack of “killing” from the host immune cells, likely due to immune evasion by HIV and dysfunctional HIV-specific T cells (Collins et al., 1998; Fenwick et al., 2019). A promising new approach to enhance the targeting and killing of HIV expressing cells is using chimeric antigen receptors (CARs) (Kuhlmann et al., 2018). T cells modified with new anti-HIV CAR technology can potentially overcome the limitations and barriers that natural HIV-specific T cells are currently facing. Compared with natural conventional effector T cells, CARs can prevent or limit viral immune escape since they directly recognize antigens irrespective of MHC presentation. CAR T cells can also be generated and allowed to expand several orders of magnitude *in vitro* or *in vivo* in a patient, which provides large numbers of engineered antigen specific cells. Ideally, CAR-expressing cells can be engineered to confer a stable and durable immune surveillance to HIV reservoirs.

However, it is still unclear how CAR-modified T cells will perform under a very low HIV antigen environment, therefore combining CAR T cell therapy with LRAs might increase CAR T cell response to latently infected cells. In this review, we summarize current developments to enhance HIV-specific CAR T cell therapy to target the HIV reservoir. In addition, we discuss how future investigation of the “kick and kill” strategy in combination with anti-HIV CAR T cell therapy can lead to synergistic effects to deplete the viral reservoir and setup a closer step to achieve a functional cure of HIV infection.

### CAR T Cells to Combat HIV Infection

The concept of using adoptive T cell therapy for treating HIV infection had been proposed decades ago. The earliest study on HIV CAR T cell therapy was designed by transferring adoptive T cells expressing a CAR that was a fusion of CD4 extracellular domain (the primary HIV cellular receptor) to the CD3ζ signaling domain (CD4ζ) (Mitsuyasu et al., 2000; Walker et al., 2000; Deeks et al., 2002). The advantage of choosing CD4 as the reactive ligand for anti-HIV CAR design is that, as the natural HIV envelope recognition moiety, CD4 ensures broad targeting of all HIV isolates. Moreover, CD4 binding sites on the envelope protein are relatively well conserved (Wang et al., 2019), as it mutation would diminish CD4 binding and have a direct effect on decreasing viral fitness. Several clinical trials were performed to test the efficiency and safety of the first-generation CD4-based CAR in HIV patients (Mitsuyasu et al., 2000; Walker et al., 2000; Deeks et al., 2002). It was demonstrated that the treatment did not result in durable control of viral replication; however, there were no overt toxicities associated with the treatment, and the modified cells persisted for >10 years (Mitsuyasu et al., 2000). The reasons for the lack of viral control could be due to several factors: (1) CD4-based CARs render the gene-modified T cells susceptible to HIV infection and elimination of activated cells, (2) lack of efficient activation signaling from costimulatory signals, (3) suboptimal T cell handling and expansion, and/or (4) lack of viral antigen stimulation. Nevertheless, the CD4ζ T cell therapy was confirmed safe and sustained stable levels of engraftment (Scholler et al., 2012).

**Figure 1:**
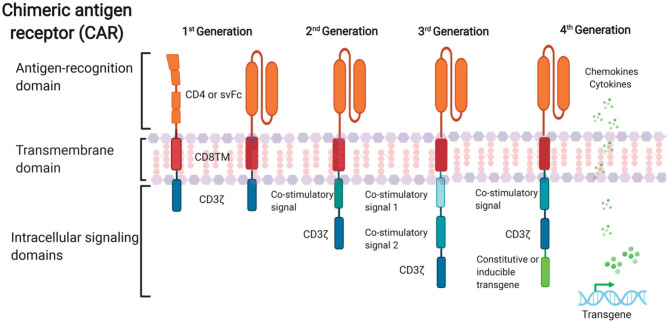
Schematic representation of anti-HIV CAR structure. First-generation CAR consists of a single anti-HIV env domain (CD4 or svFc), the transmembrane domain region, and the T cell receptor CD3-ζ domain. Second-generation CAR incorporates an additional costimulatory signaling domain to the basic first-generation receptor configuration. Third-generation contain more than one co-stimulatory domain. Fourth-generation CARs are characterized by addition of constitutive or inducible transgenes like cytokines or chemokines.

Further progress in CAR design with the aim of optimizing CAR T cell effector function and persistence in the cancer field have led to rapid advancement in CAR T cell therapy in recent years. Four generations of CARs have been developed so far (Figure 1). The first-generation of CARs linked an extracellular antigen recognition moiety to a lymphocyte-stimulating intracellular endodomain, such as the signal-transducing subunit of the TCR CD3ζ chains (Eshhar et al., 1993). First-generation CAR T cells tended to have limited *in vivo* expansion and cytotoxicity and were highly prone to apoptosis (Heuser et al., 2003; Zhao et al., 2009). The addition of costimulatory molecule domains, such as CD28 or 4-1BB, with the cytoplasmic tail of CD3ζ-containing first-generation constructs had generated second-generation CARs. Optimized anti-HIV second-generation CAR T cells that contained the costimulatory 4-1BB domain were at least 50-fold more potent at suppressing HIV replication *in vitro* than T cells expressing first-generation CARs only (Leibman et al., 2017). Animal studies also demonstrated that secondary generation CARs were superior at expanding in response to antigen, protecting CD4+T cells from HIV infection and reducing CD4 decline compared to the CAR without costimulatory molecules (Leibman et al., 2017). Further, comparable studies demonstrated that the 4-1BB costimulatory domain is superior to the CD28 domain for reducing viral rebound after ART treatment and promoting T cell persistence *in vivo* in the absence of antigen (Zhang et al., 2007; Leibman et al., 2017). Third-generation CARs were created by incorporating multiple costimulatory molecules into secondary generation CARs. Third-generation CARs have been developed with enhanced effector function, proliferation, survival, and ultimately enhanced tumor killing in the cancer field (Savoldo et al., 2011). A third-generation anti-gp120 CAR moiety, combining multiple intracellular signaling domains (CD3ζ-CD28-41BB), displayed augmented potency in lysing Env-expressing cells *in vitro* compared to the CD4ζ-CAR (Liu et al., 2016). Fourth-generation CAR T cells, known as T cells redirected for universal cytokine-mediated killing (TRUCKs), contained a third stimulatory signal which produces cytokines, such as IL-7, IL12, IL-15, or IL-18, in secreted or in a membrane-tethered form that aims to improve CAR T cells expansion and persistence and are under investigation in the oncology field to target solid tumors (Chmielewski et al., 2014; Chmielewski and Abken, 2015; Hurton et al., 2016).

Successful development and application of CAR T cell therapy in clinical cancer studies have also fostered investigations of CAR therapy into the HIV cure field. CAR therapy to target HIV reservoirs is promising for several reasons. Firstly, CAR T cells are capable of long-term immune surveillance. Effector function of CAR-modified peripheral T cells can be retained for 6 months (Kalos et al., 2011; Kochenderfer et al., 2012; Maude et al., 2014). Moreover, animal studies suggest that hematopoietic stem cell (HSPC)-derived CAR cells can persist even longer, providing continuous production of CAR T cells (Zhen et al., 2017b). Importantly, CAR T cells can be reprogrammed and differentiated into effector memory or central memory T cells. As a result, they can potentially provide long-lived immunological memory (Kawalekar et al., 2016). Secondly, CAR T cells are capable of trafficking to the different types of tissue reservoirs, including the central nervous system, which is a potentially important sanctuary for latent HIV (Marban et al., 2016). Evidence that CD19-targeted CARs can traffic to the brain and eliminate cancer supports the notion that CAR T cells may show effective against HIV reservoirs in brain tissue, where it is difficult for drugs to penetrate the blood-brain barrier (Grupp et al., 2013; Maude et al., 2014). Importantly, HSPC-based CAR expressing cells were found in multiple lymphoid tissues, including various lymph nodes, gut, and bone marrow, which are major viral replication sites in a simian-human immunodeficiency virus (SHIV) infected non-human primate (NHP) model (Zhen et al., 2017b). Moreover, CARs can also be further engineered with homing receptors to enhance CAR T cell penetration into the B cell follicle, another major viral reservoir that is difficult to target by CTLs (Haran et al., 2018). Thirdly, CAR T cells are capable of targeting antigen in a major histocompatibility complex (MHC)- independent fashion, which potentially allows therapeutic use by all. In addition, lack of MHC-restriction could make these cells less susceptible to immune escape due to viral downregulation of MHC-I in HIV infected cells (Collins et al., 1998; Goulder and Walker, 1999; Wonderlich et al., 2011). The properties discussed above could allow CAR T cells to confer stable and durable immune surveillance to HIV reservoirs if viral antigen is reactivated at these sites. Therefore, CAR T cell therapy offers a promising approach to eradicate HIV reservoirs.

### Strategies for Optimizing CARs Design Against HIV

One of the potential reasons the first CAR T cell trials for HIV infection did not result in impactful reductions in viral load is the notion that the CD4 based anti-HIV CAR design rendered the CAR-expressing T cells susceptible to direct HIV infection (Zhen et al., 2017a). In addition, antibody-based anti-HIV specific CARs could also potentially facilitate HIV infection (Leibman et al., 2017). Effective CAR-based therapeutic approaches would benefit from adding protective mechanisms in the CAR design against HIV infection. Many approaches have been developed for engineering T cells to become resistant to HIV infection utilizing an anti-HIV gene therapy reagent (Carrillo et al., 2017; Zhen et al., 2017a). These approaches have utilized small hairpin RNA (shRNA)-targeted knockdown of the HIV coreceptor CCR5 (Shimizu et al., 2010; Zhen et al., 2015) or shRNA mediated degradation of HIV RNA by targeting specific HIV long terminal repeated sequences (Ringpis et al., 2012; Kamata et al., 2015). Variations of gp41 heptad repeat 2 domain (Leslie et al., 2016), such as anti-HIV fusion peptide C46, have also been used to protect cells from infection (Younan et al., 2013; Zhen et al., 2015, 2017a). C46 has also been used in combination with the anti-CCR5 shRNA to prevent infection by dual tropic viruses (Zhen et al., 2015, 2017a). Another strategy linked CD4 portion to the carbohydrate recognition domain (CRD) of a human C-type lectin, and the bispecific CARs were completely devoid of the undesired activity of rendering CCR5+ CAR-transduced cells susceptible to HIV-1 infection. The possible reason could be CRD blocks the unwanted HIV entry receptor activity by binding to high-mannose glycans on the viral envelope spike (Feinberg et al., 2001; Ghanem et al., 2018).

In addition to CD4-ligand based CARs, antibody-based CARs can be used to target HIV infection. Most CARs currently in clinical trials for various malignancies have antibody-based antigen recognition regions (Sommermeyer et al., 2017; Guedan et al., 2019). For HIV infection, single-chain variable fragment (scFv) derived from broadly neutralizing antibodies (bNAbs) have been employed to generate HIV-specific CARs. The exponential growth in HIV bNAb identification has provided great opportunities for creating bNAb-CARs that can be potentially more effective in HIV elimination (Burton et al., 1994; Scheid et al., 2011; Huang et al., 2012; Sok and Burton, 2018). bNAb-based CARs contain scFvs derived from bNAbs, which target conserved sites within the Env protein and have been shown to broadly recognize over 95% of HIV-1 strains (Walker et al., 2011; Doria-Rose et al., 2014; Huang et al., 2016). Another study tested a panel of seven HIV-specific CARs based on well-defined HIV-1 bNAbs, which have shown variance in their breadth of HIV-1 sequence diversity coverage. Each scFv-CAR endowed CD8+ T cells with the capacity to proliferate and kill infected cells, and suppress viral replication *in vitro* (Ali et al., 2016). However, additional studies will be necessary to understand and evaluate *in vivo* prosperities and functions for bNAb-CARs.

Nevertheless, a major limitation for single antigen recognition domain-based CAR design, especially for scFV-based CARs, is a high potential for antigen escape and/or lack of antigen expression that can render the CAR T cell therapy inefficient. Recent clinical trials using monotherapy of bNAbs 3BNC117 (Scheid et al., 2016) or VRC01(Bar et al., 2016) showed a decrease in plasma viremia and a delay, but not a prevented viral rebound. However, treatment with a combination of two or more bNAbs can significantly reduce the viral reservoir and showed prolonged viral suppression (Bar-On et al., 2018; Mendoza et al., 2018). Thus, a single bNAb CAR might not be sufficient to suppress HIV-1 for the long term since escape mutants that emerge *in vivo* will allow viral rebound. The use of a combination of antigen recognition domains in multiple CARs or the use of a CAR with multiple antigen recognition domains would likely provide far greater suppression. Approaches utilizing multiple antigen recognition domains are currently under development, termed dual-, bi-, or tri-specific CARs (Ruella et al., 2016; Fry et al., 2018). Dual or bispecific CARs have been recently developed that connect an extracellular CD4 domain to either a bNAb-based scFv (Liu et al., 2016; Anthony-Gonda et al., 2019) or the CRD of a human C-type lectin (Ghanem et al., 2018; Haran et al., 2018). These have been shown to have antiviral effects in redirecting T cells *in vitro* to kill HIV infected cells and, in some cases, *in vivo* in model systems; however, their effects *in vivo* in humans are not well characterized.

In addition to CAR T cell-mediated immunotherapy, an anti-HIV CAR-based approach with natural killer (NK) cells has also been considered as a strategy for antiviral immunotherapy. A key feature of NK cells is that they, in addition to T cells, express the intracellular signaling machinery to allow a CAR expressing the CD3ζ signaling domain to function in redirecting killing activity to the antigen of interest (Liu et al., 2017; Mehta and Rezvani, 2018). Unique anti-HIV features of NK cells makes them an attractive and effective tool for immunotherapy. HIV nef-mediated MHC-1 downregulation can potentially expose the HIV infected cells to be more susceptible to NK cells lacking inhibitory receptors to HLA-C and HLA-E (Bonaparte and Barker, 2004). Moreover, HIV-infected target cells can increase the expression of ligands, such as unique long binding protein ULBP-1 and−2, which can be recognized by NK cells (Richard et al., 2010). These upregulated ligands can induce NK activating receptor (natural killer group 2, member D, NKG2D) -mediated effector functions such as cytotoxicity and cytokine production in human and mouse NK cells (Ogasawara and Lanier, 2005; Bryceson and Ljunggren, 2008; Le Bert and Gasser, 2014; Stojanovic et al., 2018). T follicular helper cells (Tfh cell), a critical reservoir that is established during HIV infection, can also be eliminated by NK cells (Rydyznski et al., 2015). In addition, NK cells can identify and remove HIV infected cells through antibody-dependent cell-mediated cytotoxicity (ADCC) mechanism (Chung et al., 2008, 2011). Therefore, these properties make genetically modified NK-cells an appealing tool to be tested as a novel strategy to control HIV replication and reduce HIV reservoir by directing them to HIV through the use of a CAR. Clinical trials are ongoing using CAR-modified NK cells for cancer immunotherapy and the safety and efficacy of these therapies will be evaluated. A Phase I clinical trial was completed using CD19-41BB-TCRζ CAR NK cells to target B-lineage acute lymphoblastic leukemia (ALL) malignancy (NCT00995137). Another Phase II clinical trial with NK cells has been approved for B-lymphoid malignancies using a CD28-TCRζ CAR with an inducible suicide gene and an activating cytokine (NCT03056339). Recently, this Phase II trial reported that 73% of patients exhibited a clinical response without the development of major toxic effects after administration of NK cells engineered with a CAR targeting CD19 and co-expressing IL-15 (Liu et al., 2020). These trials suggest a promising aspect in the use of redirected immunity using CAR-NK cells.

In early research of NK-based CAR against HIV, CD4-TCRζ CAR-modified NK cells were shown to effectively kill either NK-resistant tumor cells expressing the relevant ligand, gp120, or CD4+ T cells infected with HIV *in vitro* (Tran et al., 1995). We later demonstrated *in vivo* in humanized mice that CAR-modified HSPCs can differentiate into multiple hematopoietic lineages, including functional NK cells (Zhen et al., 2015). These NK cells are resistant to HIV infection and may have played a role in suppressing HIV replication. CAR-modified NK cells derived from CAR-HSPCs were also detected in NHP models (Zhen et al., 2017b). CAR-modified HSPCs appear to possess the ability to produce functional CAR NK cells continuously over time, overcoming one of the limitations of NK cell-mediated immunotherapy in the relatively short life span of NK cells isolated directly from patient's peripheral blood (Liu et al., 2017). The production of CAR NK cells from HSPCs in addition to T cells could provide added immune surveillance benefits by targeting different tissues and reservoirs and by supplying a different type of cellular immunity.

### Strategies to Enhance CAR T Cell Engraftment, Function, and Persistence

The use of CAR or TCR modified autologous peripheral blood T lymphocytes for B-lineage malignancies therapy has shown tremendous clinical success (Ramos et al., 2016; June et al., 2018). However, it remains unclear if CAR T cells can respond to malignancies that may recur after treatment, as antitumor activities of these cells appear to diminish over time (Mueller et al., 2017). As described above, there was limited functionality in autologous peripheral CAR T cells in suppressing HIV in past clinical trials. These studies revealed that a CAR T cell approach is safe and feasible and demonstrated the persistence of gene-modified T cells for years after infusion. However, the reasons for the long-term maintenance of this (albeit small) population of cells are not clear and could be due to homeostatic persistence mechanisms. To achieve a sustainable virologic control after ART cessation, strategies to enhance CAR-expressing cell engraftment, function, and persistence *in vivo* seem to be required.

Strategies to enhance CAR T cell function and persistence have been thoroughly investigated for CD19-based and other tumor-specific antigen targeting CARs (Fesnak et al., 2016; Labanieh et al., 2018). For example, the use of cytokines IL-7 and IL-15 for culturing and expanding CD19CAR T cells resulted in superior expansion and generation of naïve and memory populations that resulted in better persistence and antitumor efficacy *in vivo* compared to CD19CAR T cells cultured in IL-2 alone (Xu et al., 2014; Zhou et al., 2019). The endogenous expression of cytokines by CAR-expressing T cells has also been evaluated to boost persistence and efficacy *in vivo*. A vector containing a CD19CAR co-expressing IL-15 and the suicide gene iC9 has been evaluated in a preclinical study (Hoyos et al., 2010). The iC9/CAR.19/IL-15 not only persisted better but also had greater antitumor efficacy *in vivo* compared to CD19CAR. In addition, the IL-15 had other beneficial effects such as greater expansion, lower cell death, and lower programmed cell death-1 (PD-1) expression in response to antigen stimulation. Other cytokines such as IL-12, IL-7, IL-21, and IL-18 have found to have similar improved antitumor effects (Chinnasamy et al., 2012; Pegram et al., 2015; Hu et al., 2017; Adachi et al., 2018; Batra et al., 2020). Whether culturing conditions, endogenous expression, or administration of immunostimulatory cytokines have similar effects *in vivo* for HIV-specific CARs has yet to be determined. It is cautionary to use such cytokines in HIV targeted therapies during infection and/or in the absence of ART as some of these cytokines, specifically IL-7 and IL-15, may boost immune activation which can promote virus production and lead to higher levels of viremia (Managlia et al., 2006; Mueller et al., 2008; Vassena et al., 2012; Coiras et al., 2016; Manganaro et al., 2018). Another approach to boost CAR T cell function *in vivo* is by blocking immune checkpoint molecules. Strategies to block PD-1 by CRISPR, shRNAs, or PD-1 antibody blockade have been implemented and tested into cancer-specific CARs and observed to improve antitumor responses *in vivo* (Cherkassky et al., 2016; Rupp et al., 2017; Rafiq et al., 2018; Hu et al., 2019a,b). Although PD-1 blockade has been tested in HIV infection to improve T cell responses and suppress viremia, it remains to be seen whether endogenously blocking PD-1 or other immune checkpoint molecules will boost HIV specific CAR T cell responses *in vivo* (Palmer et al., 2013; Seung et al., 2013). Overall, many strategies to improve peripheral CAR T cell function and persistence *in vivo* have been tested and confirmed in the cancer field. However, it is not yet clear whether any of these strategies will show similar outcomes with HIV-specific CARs.

Another approach, as mentioned above, to solve issues with engraftment, function, and persistence is to engineer the expression of CARs in an HSPC-based approach. Despite the adaptation of improved T cell handling techniques and inclusion of anti-HIV reagents, we found that CD4-CAR T cells made from peripheral blood T cells persisted at low levels and had limited antiviral effects in HIV-infected humanized mice (unpublished data). In contrast, proof of concept studies conducted in our group demonstrated that HSPCs are capable of lifelong engraftment and allow normal development of CAR T cells *in vivo* (Kitchen et al., 2012; Zhen and Kitchen, 2013; Zhen et al., 2017b). This includes thymic selection, eliminating potentially self-reactive T cells, and increasing the potential for the development of immunological memory (Kitchen et al., 2012; Zhen and Kitchen, 2013; Gschweng et al., 2014). Most importantly, our previous data showed that autologous HSPCs modified with a TCR molecular clone (Kitchen et al., 2012) or CD4-CAR (Zhen et al., 2015) against HIV resulted in successful T cell differentiation and significant suppression of HIV replication in humanized bone-marrow-thymus-live mice (huBLT). Further, we demonstrated the feasibility, safety, and potential efficacy of the overall HSPC-based CAR approach in NHPs (Zhen et al., 2017b). In the NHP study, we observed normal hematopoietic recovery, and long-term maintenance of CAR-modified cells (over 2 years) in the absence of any adverse events such as oligoclonal expansion of cells, cytokine storms, self-reactivity, or any other health alterations in transplanted animals. Importantly, we found that CAR-HSPCs transplanted animals have a reduced magnitude of rebound viremia after ART cessation as compared to controls (Zhen et al., 2017b). CAR cells were found in multiple lymphoid tissues, resulting in decreased viral RNA levels in tissues and protection of CD4+ T cells in the gut, which is one of the primary replications and reservoir sites for HIV. Moreover, CAR-engineered HSPCs in both huBLT and NHP models can produce myeloid and NK cells in addition to T cells expressing the CAR, suggesting that CAR-engineered immune cells derived from HSPCs can provide broader immune responses to HIV reactivation after ART interruption. Therefore, stem cell-based CAR offers a promising approach to generate long-term and effective anti-HIV immunity.

### Improving CAR Design and Targeting the HIV Reservoir

Several groups have been studying new generations of CAR T cell therapies and their effects on targeting the HIV reservoir. One study engineered potent bNAb-based single-chain variable fragments fused to second-generation CAR signaling domains (Hale et al., 2017). bNAb-based CAR T cells showed specific activation and killing of HIV-infected vs. uninfected cells in the absence of HIV replication. The study also demonstrated that homology-directed recombination of the CAR gene expression cassette into the *CCR5* locus enhances the suppression of replicating viruses compared with CAR expression alone. Therefore, this work suggested that HIV immunotherapy utilizing an approach that directly delivered the CAR into the *CCR5* locus of T cells by homology-directed gene editing is feasible and effective. A CD4-based CAR T cell therapy with CCR5 disruption by zinc-finger nucleases is in phase I clinical trials to treat HIV and examine effects on the reservoir (NCT03617198).

A third-generation anti-HIV CAR molecule (CD3ζ-CD28-CD137) has been developed that consists of a scFv region derived from the bNAb VRC01 capable of redirecting the antigen specificity of primary CD8+ T cell populations against gp120 (Liu et al., 2016). Interestingly, the bNAb-based CAR T cells were able to effectively kill the reactivated HIV-infected CD4+ T lymphocytes isolated from HIV-infected individuals receiving cART, suggesting CAR T could be a potential therapeutic strategy to eradicate HIV (Liu et al., 2016). Moreover, this research is entering clinical trials (NCT03240328) to evaluate bNAb (VRC01)-based CAR for latent reservoir eradication.

Another strategy to improve targeting of HIV reservoir is to modify bispecific CARs with a homing chemokine receptor CXCR5. CD4+ CXCR5+ TfH cells in B cell follicles of lymphoid tissue have been reported to represent a major HIV reservoir compartment harboring intact and infective proviruses (Perreau et al., 2013; Banga et al., 2016). However, HIV-specific CTLs that recognize and kill virus-producing T cells are found in low numbers within the follicle due to reduced expression of CXCR5 (Mylvaganam et al., 2017; Reuter et al., 2017). Thus, the expression of CXCR5 on HIV-specific CAR T cells might promote their homing to lymph nodes to target latently infected TfH cells. Recent research successfully designed a bispecific anti-SIV CAR co-expressing the rhesus macaque follicular homing chemokine receptor CXCR5 to enhance CAR T cell trafficking to B cell follicles. The functionality of the CAR/CXCR5 T cells was demonstrated through their potent suppression of SIV replication *in vitro* and migration to B cell follicles in lymphoid tissues *ex vivo* (Haran et al., 2018).

Most recently, a universal CAR T cell platform, *convertible*CAR T cells, was designed to redirect CTLs by binding a broadly neutralizing anti-HIV antibody or antibodies. *convertible*CAR was modified with the NKG2D receptor, which can turn the T cell into a potent killer, but only when bound to its partner MIC (MHC-class I-like Complex, natural ligands for NK2G)-bNAbs. *convertible*CAR T cells effectively kill HIV-infected, but not uninfected, CD4 T cells from blood, tonsil, or spleen and only when armed with anti-HIV bNAbs. *convertible*CAR T cells can also result in a 50% reduction in the amount of HIV RNA expression in cultured T cells derived from HIV-infected individuals after reactivation (Herzig et al., 2019). The modularity of *convertible*CAR T cell system, which allows multiplexing with several anti-HIV antibodies yielding greater breadth and control, makes it a promising tool for attacking the latent HIV reservoir.

Another recent study engineered T cells with up to three functionally distinct HIV envelope-binding domains to form bi-specific or tri-specific targeting anti-HIV CAR T cells. Three putative targets included the gp120 CD4-binding site, gp120 coreceptor–binding site, and gp41 near the membrane-proximal external region. Bi-and tri-specific CAR T cells showed the capacity to potently reduce cellular HIV infection both *in vitro* and *in vivo*. The multi-specific CARs efficiently killed HIV-infected cells in a humanized mouse model while protecting the CAR T cells from genetically diverse HIV infection (Anthony-Gonda et al., 2019). Despite the lack of evidence whether multi-specific CAR T cells can effectively migrate to a variety of different tissue sites where established HIV reservoir exist and whether they can target HIV latently infected cells after ART interruption, these data strongly support multi-specific anti-HIV CAR as a promising approach for HIV functional cure.

In spite of the recent success of CAR T therapies in targeting HIV, none of the research has shown an effective and significantly durable reduction in HIV viral load after the adoptive transfer of CARs in HIV/SIV infected animals. None of the recent studies so far have tested different types of new generation CAR T cells in killing reactivated HIV *in vivo*. Thus, further research on the capacity of new generation CARs to eradicate the HIV reservoir should be evaluated in HIV/SHIV infected animal models. The application of CAR therapy in HIV cure strategies is just beginning to be explored, and more work is needed. Better designed CARs should also be considered to increase the cytotoxicity/efficacy, improve the proliferation/persistence, prevent exhaustion/senescence, and lower the potential for resistance/escape.

### “Kicking” the HIV Reservoir for CAR T Cell “Killing”

**Figure 2:**
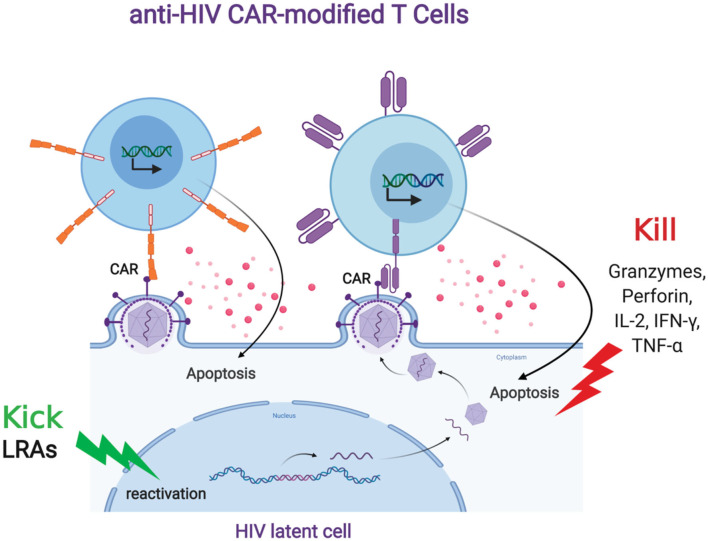
Anti-HIV CAR modified T cells and the “Kick and Kill” strategy to eliminate HIV latently infected cells. The “kick” strategy uses LRAs to induce HIV transcription, protein expression, and virion production. The CD4 (top left, orange) or antibody (top right, purple) based CAR engineered T cell targets an HIV binding site on a viral particle on the cell surface of a reactivated reservoir cell (bottom). Upon binding, the CAR modified T cell will release granzymes and cytokines to “Kill” the HIV infected cells.

HIV persistence despite ongoing, long term antiretroviral therapy is largely due to the ability of the virus to latently persist in various anatomical reservoirs. Targeting these reservoirs by any immune surveillance mechanism is difficult due to the lack of viral antigen expression. There are several strategies that aim to induce latent viral expression to allow immune targeting and elimination. One such strategy, known as the “kick and kill” approach, seeks to induce the virus out of latency to allow immune-mediated killing. The current paradigm for “kick and kill” strategies to eliminate the HIV reservoir involves the transcriptional reactivation of the integrated provirus in latently infected cells and allow the viral antigen to be presented to immune surveillance in ART-treated individuals. CAR T cells can be an optimal “kill” response in the “kick and kill” strategy (Figure 2). However, this will depend on robust HIV expression from latently infected cells; therefore, a combinatorial therapy with potent latency reversing agents (LRAs) will be necessary to effectively eradicate the reservoir (Bashiri et al., 2018). LRAs have been tested in animal models and clinical trials and shown to induce HIV expression and is well tolerated *in vivo* (Marsden et al., 2017; Thorlund et al., 2017). However, clinical studies have not shown that LRAs alone can significantly decrease the viral reservoir. A likely explanation for this is that the host immune response present in these sites is dysfunctional and incapable of effectively clearing the virus (Collins et al., 1998; Appay et al., 2000; Day et al., 2006; Trautmann et al., 2006; D'Souza et al., 2007; Buggert et al., 2014; Huang et al., 2018). CAR-mediated redirection of T cells and other immune cells could provide greater numbers of HIV-specific cells with a greater functional capacity to eliminate virus-reactivated cells. Another possible reason CTLs at sites of virus reactivation are not effective in killing HIV infected cells could include Nef mediated immune evasion by downregulation of HLA class I molecules. CAR T cells have the advantage of overcoming this type of immune evasion because of its non-dependence in the use of HLA molecules to recognize HIV envelope. Another potential advantage of CAR T cell is that they will more likely target latently infected cells that express provirus with intact envelope rather than defective provirus with envelope deletions, which natural CTLs will target (Bruner et al., 2016; Pollack et al., 2017; Huang et al., 2018). A key question will pertain to the optimal combination of types of LRAs that will induce the minimal levels of HIV expression required for CAR T cell recognition and killing. Ideally, the best LRAs to aid CAR T cell therapy to target the reservoir will be those that will potently induce HIV expression and innate immune response that can provide cytokine support for CAR T cells without major side effects *in vivo*. However, CAR-expressing cells offer a potentially more effective “Kill” component in this overall approach.

A key issue in the “kick and kill” strategy is finding an optimal LRA that allows effective virus reactivation in the absence of gross immune activation and side effects such as cytokine storms. Many different LRAs are currently under investigation and could complement the use of CAR-based approaches in targeting the HIV reservoir. LRAs that are classified as histone deacetylase (HDAC) inhibitors (HDACi) have been the most studied in clinical trials (Rasmussen and Lewin, 2016). Although HDAC inhibitors had minimal impact on reducing the size of the viral reservoir in these trials, the drugs Vorinostat, Panobinostat, and Romidepsin did result in measurable increases in the cell-associated HIV RNA levels in CD4+ T cells (Archin et al., 2012, 2017; Elliott et al., 2014; Rasmussen et al., 2014) and plasma viremia in patients (Rasmussen et al., 2014; Sogaard et al., 2015). Thus, because of their safety and induction of latency reversal in several clinical trials, these drugs may be prime candidates to combine with CAR T cell therapy and study the effects on the viral reservoir. Another class of small molecules that have been effective in potently inducing HIV expression are protein kinase C agonists (PKCa) (Williams et al., 2004; Bullen et al., 2014; Jiang and Dandekar, 2015; Laird et al., 2015; Brogdon et al., 2016). The PKCa bryostatin-1 has been tested in phase I clinical trial in ART suppressed patients (Gutierrez et al., 2016). The results of this study showed no impact on the induction of HIV expression, most likely due to a single round of low doses that was well tolerated. However, a study using a humanized mouse model testing synthetic bryostatin analogs showed promising “kick and kill” results (Marsden et al., 2017). The bryostatin analog induced T cell activation in blood and spleen. ART-treated mice that received a single dose of bryostatin analog showed a significant increase in virus expression in blood and spleen and an increase in cell death compared to the control group. The synergistic effect of combining PKCa ingenol-B with HDACi vorinostat was seen in 1 out of 2 ART-treated SIV infected animals that resulted in detectable viral load in plasma and cerebrospinal fluid, which continued to increase several days after LRA interruption (Gama et al., 2017). Other studies using a combination of PKCa and HDAC inhibitors have found similar synergetic results and latency reversal in different memory CD4+ T cell subset populations (Darcis et al., 2015; Laird et al., 2015; Pardons et al., 2019). The combination of HDAC inhibitors and PKCas may provide a more robust “kick” in latency reversal that, in combination with CAR T cells, can potentially lead to a greater killing of the latent reservoir.

Another class of LRAs that have shown to potently induce latency reversal are Toll-like receptor (TLR) agonists (Macedo et al., 2018). In particular, TLR-7 and TLR-9 agonists have shown to be effective HIV latency inducers (Offersen et al., 2016; Tsai et al., 2017). A clinical trial has tested a TLR-9 agonist MGN1703 in virus suppressed individuals (Vibholm et al., 2017). The TLR-9 agonist was well tolerated and led to activation of plasmacytoid dendritic cells, increased activation of NK cells and CD8 T cells. Increased plasma HIV RNA levels were seen only in some of the participants; however, no effect on viral reservoir size was observed. Likewise, with TLR-9 agonists, TLR-7 agonists have also been found to induce viremia, induce activation of CD8+ T cells and NK cells, and target the reservoir in NHPs (Lim et al., 2018). Remarkably, 2 of 9 animals treated with the TLR-7 agonist remained aviremic for over 2 years after ART interruption. In a different study, TLR-7 agonist GS-986 treatment alone led to activation of CD8+ and CD4 T+ cells along with innate immune stimulation (Borducchi et al., 2016). However, a combination treatment using GS-986 (TLR7 agonist) and an Ad26/MVA vaccine led to a significant delay in viral rebound after ART interruption. TLR agonists can potentially be used in combination with CAR T cells to stimulate innate immune cells and support CAR T cells with stimulating cytokines that may lead to increased persistence and expansion, which can result in a synergistic eradication effect.

Cytokines such as IL-2 and IL-7 were initially investigated as potential LRAs to induce HIV expression and target the reservoir (Chun et al., 1999; Stellbrink et al., 2002; Wang et al., 2005; Levy et al., 2012). Recently, Interleukin-15 (IL-15) has also been investigated as a promising target to reactivate the HIV virus and control viral replication (Jones et al., 2016; Ellis-Connell et al., 2018). An IL-15 superagonist ALT-803 was found to induce HIV RNA production in PBMCs obtained from ART-treated participants and enhanced killing of HIV infected cells induced by the superagonist (Jones et al., 2016). More recently, in a NHP model, superagonist IL-15 S-803 induced reactivation of SIV virus but only after depletion of CD8+ T cells, thus revealing a role of CD8+ T cells blocking latency reversing effects of N-803 (McBrien et al., 2020). In addition to the LRA effects of IL-15 superagonist, another study using SIV infected NHP animals has shown that IL-15 ALT 803 can direct SIV specific CD8+ T cells into B cell follicles, which led to decreased SIV RNA or SIV DNA harboring cells in lymph nodes after treatment (Webb et al., 2018). Whether the IL-15 superagonists have the same effects in humans remains to be seen. However, a report from a phase I clinical trial using IL-15 ALT-803 on HIV infected ART-treated participants so far has shown that the drug is well-tolerated, increased NK, CD4+, and CD8+ activation in lymph nodes, and increased HIV transcription following initial treatment was observed (Zachary et al., 2018). Overall, IL-15 superagonists can be used as LRAs with the additional beneficial immune activation properties that, if combined with CAR T cells therapy, can lead to improved trafficking of CAR T cells to reservoir sites *in vivo*.

Although the strategy of combining LRAs that target multiple pathways to induce HIV expression with CAR T cell therapy to purge the viral reservoir can be promising, a current major limitation of LRAs is the inability to reactivate a significant portion of the latent reservoir. To overcome this limitation, other strategies are needed to eliminate unresponsive HIV harboring cells to LRAs. Strategies that target the provirus and attempt to specifically excise it from these cells are under development. Genome editing using CRISPR-Cas9 has been tested *in vivo* using animal models to permanently remove HIV provirus from the genome and shown promising results (Kaminski et al., 2016; Yin et al., 2017; Wang G. et al., 2018; Dash et al., 2019). However, there is a current limitation using CRISPR-Cas9 as HIV can escape through mutations targeted by the single guide RNA (sgRNA) (Wang G. et al., 2016; Wang Z. et al., 2016, 2018). Alternatively, CRISPR-Cas9 system can be used to induce HIV expression by using a catalytically inactive Cas9 fused with a transcriptional activator (Limsirichai et al., 2016). Using this CRISPR-Cas9 transcription activation system, the authors found that combining it with the HDACi suberoylanilide hydroxamic acid (SAHA, also known as Vorinostat) and a prostratin molecule can synergistically increase HIV expression using J-lat cell lines *in vitro*. It is yet to be seen whether this approach can reactive HIV *in vivo* without any toxic side effects. In summary, CAR T cell therapy can provide the immune surveillance to “kill” latently infected cells in response to agents that can induce HIV expression and innate and cellular immune responses that can support CAR T cell cytotoxicity and trafficking to tissues that harbor viral reservoir.

A major challenge for LRAs to target and reactivate all latent cells is the heterogeneity of the reservoir. The diverse response of latent cells to LRAs have been shown to be based on several factors including cell type, silencing mechanisms inhibiting HIV, tissue reservoir, and patient gender (reviewed in Ait-Ammar et al., 2019). Treatment of CD4+ T cells from ART-treated patients with PKC agonists bryostatin and ingenol revealed that bryostatin was effective in reactivating T effector memory (T_EM_) cells whereas ingenol was more effective in reactivating HIV in both T central/transitional memory (T_CM/TM_) and T_EM_, suggesting that T cell memory subsets harboring HIV may not be equally susceptible to the same class of LRAs (Baxter et al., 2016). More recent studies are showing similar results with other classes of LRAs and highlighting the heterogenous responses to several LRA families among different CD4+ T cell subsets; thus, implicating a rationale for the use of combined LRAs to overcome the heterogeneous reservoir (Grau-Expósito et al., 2019; Kulpa et al., 2019). Determining the right combination of LRAs to reactivate these reservoirs will be key to study the effectiveness of anti-HIV CARs, whether it be CD4-based, bi-specific, or tri-specific CARs to kill the reactivated reservoirs. It is likely that cells expressing these different types of CAR molecules would have different effects on clearing out reactivated HIV envelope expressing cells, based on their antigen recognition coverage and the certain limitations that are highlighted above. A CD4-based CAR may provide the widest range of reactivated envelope recognition, but antibody-based CARs, either using multiple single CARs or CARs with multiple specificities could provide sufficient coverage and prevent immune escape. However, it remains to be seen whether anti-HIV CAR expressing cells will be able to target not just CD4+ T cell reservoirs but other cell types such as hematopoietic stem cells and cells from the myeloid lineage that can migrate to anatomical sanctuaries such as the central nervous system (CNS) (Gras and Kaul, 2010; Williams et al., 2014; Gianella et al., 2016; Zaikos et al., 2018). The CNS is considered a sanctuary site that harbors HIV infected cells that can be latently infected, including astrocytes, microglial cells, and perivascular macrophages (Thompson et al., 2011; Wallet et al., 2019). Therefore, it will be important to develop LRAs that can reactivate HIV from CNS cell types. The PKC agonist bryostatin-1 is a promising LRA to target microglial cells and astrocytes (Darcis et al., 2015; Díaz et al., 2015). Information on whether CAR-modified T cells can traffic to the CNS has recently come from clinical studies of B cell malignancies being treated with CD19CAR-modified T cells. These studies show the presence of CD19 CAR-modified T cells in the cerebral spinal fluid (CSF), suggesting these CAR cells may cross the blood brain barrier (Kochenderfer et al., 2017; Santomasso et al., 2018). In addition, a gliospecifc CAR was also found to traffic and target brain tumor in patients (O'Rourke et al., 2017). It has yet to be determined whether anti-HIV CAR T cells will be able to cross the blood brain barrier and target reactivated CNS reservoir cells. However, in our previous study using a stem cell based CD4-based CAR approach in a NHP model of SHIV infection, we observed CD4CAR gene marking in the brain tissue and, compared to control animals, CD4CAR animals showed lower SHIV RNA levels in this compartment (Zhen et al., 2017b). This suggests that, at least in a stem cell CAR model, stem cell derived CD4CAR+ cells can monitor and reduce viral burden in the brain.

Careful consideration should be given about using certain LRAs with CAR-T or CAR-NK cells, as the lack of specificity by LRAs may negatively impact T cell or NK function (Jones et al., 2014; Clutton et al., 2016; Garrido et al., 2016; Pace et al., 2016; Walker-Sperling et al., 2016; Clutton and Jones, 2018; Desimio et al., 2018). In particular, *in vitro* studies show that HDACi panobinostat is found to be toxic to NK cells, decreases their cytotoxicity, antiviral activity, cytokine production, and viability (Garrido et al., 2016). The PKC agonist bryostatin-1 was found to impair NK mediated cytotoxicity, ADCC activity, and clearance of reactivated latently infected CD4+ T cells *in vitro* (Desimio et al., 2018). In T cells, the HDACi romidepsin, panobinosat, and SAHA all found to impair cytokine production and killing of HIV infected CD4+ T cells in CTLs *in vitro*, with the greatest impairment seen with romidepsin (Jones et al., 2014). Similarly, in another study, panobinosat impaired CTL cytotoxicity, whereas romidepsin reduced viability and both impaired proliferation responses in CTLs isolated from HIV infected individuals, although the effects were dependent on exposure time (Clutton et al., 2016). The combination of romidepsin and bryostatin-1 significantly diminished the ability of HIV-specific CD8+ T cells isolated from elite controllers to suppress HIV replication (Walker-Sperling et al., 2016). Thus, some LRAs can have a negative impact on immune function of T and NK cells which may potentially impact CAR-modified T cells and NK cells. More studies are needed to evaluate the optimal doses and combination of LRAs that will reverse latency without toxicity to effector cells that directly kill HIV infected cells. Additionally, a more optimal approach can be to use a cocktail of LRAs that includes immunomodulatory LRAs such as IL-15 agonists and TLR agonists that can boost the immune response and counteract the immunosuppressive side effects of other LRA compounds.

## Conclusion

There is a considerable amount of effort underway to develop new and novel strategies to eradicate persistent HIV infection. CAR-based approaches represent a promising strategy to enhance the antiviral cellular immune response against HIV in hopes of eradicating the virus. A combination of approaches will likely be necessary to readily facilitate the successful use of anti-HIV CAR therapy to help establish long-term immune surveillance and kill the reactivated HIV infected cell. However, several issues remain to be answered for further advancement in this field: (1) Whether reactivated antigen expression induced by LRAs is strong enough to be recognized by CAR-modified immune cells? (2) Whether CAR therapy and LRAs could have effects in different anatomical tissue reservoirs, including the gut and the brain? And (3) Whether repeated CAR-modified immune cell infusion and/or repeated rounds of LRA reactivation is needed? There is a high level of optimism that the next generation CAR T cell therapy as part of the “kick and kill” regimen, in combination with other therapies such as LRAs or bNAbs, could eradicate the persistent HIV reservoir by enhancing the immune surveillance and maintaining a long-lasting viral suppression after ART interruption.

## Author Contributions

All authors listed have made a substantial, direct and intellectual contribution to the work, and approved it for publication.

## Conflict of Interest

SK is a founder of CDR3 Inc. The remaining authors declare that the research was conducted in the absence of any commercial or financial relationships that could be construed as a potential conflict of interest.
